# Comparison of quality characteristics of reconstituted glutinous rice flour and sweet dumplings with various beans

**DOI:** 10.1002/fsn3.4438

**Published:** 2024-08-29

**Authors:** Sun Qiangqiang, Liu Xuhua, Yan Rong, Wang Junying, Yang Liping, Dong Qiyun, Zhai Ligong

**Affiliations:** ^1^ Food Engineering College Anhui Science and Technology University Fengyang China; ^2^ Forestry College Nanjing Forestry University Nanjing China; ^3^ Food Processing Research & Development Center Bengbu Brothers Cereals and Oils Food Science and Technology Co., Ltd Bengbu China

**Keywords:** glutinous rice flour, heat–moisture treatments, sweet dumpling, various bean

## Abstract

This study blended five types of beans—florid kidney bean, red adzuki bean, chickpea, black bean, and white kidney bean—with glutinous rice flour (GRF) to create a synergistic heat–moisture treatment (HMT). We investigated the effects of this combination on the digestibility of the glutinous rice mixture and the quality of sweet dumplings. The inclusion of beans, along with HMT, altered the granular morphology, adhesive qualities, thermal characteristics, crystallinity, and protein secondary structure of the blended powders compared to GRF. Notably, the starch granules in the glutinous rice flour with red adzuki bean (R‐GRF) were more complete in morphology, compact in structure, and exhibited higher heat stabilization and crystallinity, with significant changes in protein secondary structure. These modifications resulted in a low expected glycemic index (eGI) value of 52.01. Additionally, the quality assessment of the fast‐frozen sweet dumplings showed that those made with red adzuki bean mixed with glutinous rice flour (R‐SD) had superior texture, reduced cracking, and less water loss compared to those made with GRF (G‐SD), while maintaining similar color and odor to G‐SD. The eGI value of 52.21 for R‐SD also fell within the low eGI range, indicating its potential for developing low‐eGI foods for patients with T2DM and other special populations.

## Graphical abstract

In order to solve the problem of the high GI value of glutinous rice flour, this experiment mainly focuses on the production of low GI value mixed flour by adding legumes to glutinous rice flour in a synergistic heat–moisture treatment. The characterization properties of starch in mixed flour were then explored through a series of experiments to determine the factors that may influence the reduction of GI values of glutinous rice flour and to analyze the quality characteristics of sweet dumplings developed with mixed flour.

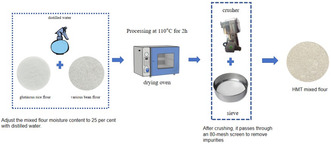

## 1 INTRODUCTION

According to survey statistics, the global prevalence of diabetes mellitus has been estimated at 463 million, with more than 90% of these cases classified as type 2 diabetes mellitus (T2DM). Among these, China accounted for approximately 20.8% of T2DM patients, representing one of the highest proportions worldwide (Majety et al., 2023). One of the factors contributing to the significant increase in diabetes was the widespread consumption of refined carbohydrates and highly processed foods (Popkin et al., 2012). These foods were often high in carbohydrates and had a high expected glycemic index (eGI), which could make glycemic control difficult, particularly for individuals with type II diabetes and others with special glycemic control needs (Paradis & Chiolero, 2011). Studies such as those conducted by Björck et al. (2000) had demonstrated that low‐eGI foods (e.g., low‐eGI cookies, pasta, and bread) were effective in slowing the rate of blood glucose elevation, thereby aiding in long‐term glycemic control. However, these low‐eGI foods did not fit into the traditional Chinese culture of staple foods. The Chinese diet had been structured around rice‐based foods such as rice, glutinous rice, and sweet dumplings (Wang et al., 2019). Sweet dumplings, made from glutinous rice flour and water, had a high overall eGI, rendering them unsuitable for T2DM patients and special populations. Consequently, the development of low‐eGI glutinous rice products suitable for T2DM patients had significant market potential and health promotion value.

Glutinous rice flour had been a high‐carbohydrate food, primarily composed of branched‐chain starch. The overall double‐helix structure of branched starch exhibited a high degree of branching and abundant amylase interaction sites. Consequently, the hydrolysis of glutinous rice starch was high, and the glucose release rate was rapid, resulting in a relatively high eGI value (Guo et al., 2015). To reduce the eGI value of glutinous rice flour, it was necessary to consider the modification treatment of glutinous rice starch to enhance its resistance to amylase and decrease the hydrolysis rate. Starch modification methods were categorized into physical modification, chemical modification, and enzyme modification (Sinhmar et al., 2023). In food processing, chemical modifications can result in residual chemical reagents, while enzyme modification methods tend to be more costly. Therefore, physical modification methods are often preferred due to their lower cost, enhanced safety, and suitability for mass production. Physical modification methods can be categorized into dry heat treatment (DHT), heat–moisture treatment (HMT), microwave treatment (MT), and high‐pressure treatment (HPT). Among these, HMT is particularly effective in significantly enhancing the RS content and notably reducing the eGI value of starch. HMT can significantly reduce the swelling degree of starch granules and increase the interaction forces between them (Tan et al., 2017). This process effectively hinders amylase from penetrating the starch interior, thereby reducing the contact sites for amylase (BeMiller & Huber, 2015). Consequently, HMT increases the RS content and decreases the eGI value (Wu et al., 2022). However, it is challenging to reduce the high eGI of food to a low‐eGI value through a single physical modification method. According to the research conducted by Nayak et al. (2014) and Saragih et al. (2020), the eGI values of potato products and wheat products can be significantly reduced by incorporating various grains.

Grains in the omnivorous food group that can significantly reduce the eGI value of foods include quinoa, buckwheat, and beans. The study by Yang et al. (2018) demonstrated that beans contain gallic acid, ferulic acid, catechins, and p‐hydroxybenzoic acid, which have an inhibitory effect on postprandial glucose elevation. Moreover, beans are rich in active substances such as polyphenols, flavonoids, and functional proteins, which can effectively inhibit the hydrolysis of α‐amylase and reduce the glycemic index (GI) value of starchy foods (Zheng et al., 2020). However, there have been relatively few studies on combining beans with glutinous rice flour to make sweet dumplings, likely due to the presence of fat‐oxidizing enzymes in beans. This enzyme can oxidize polyunsaturated fatty acids during processing, resulting in the formation of small molecular alcohols, aldehydes, ketones, and other volatile compounds with various off‐flavors, which can significantly affect the qualities associated with sweet dumplings (Zhang et al., 2015). Therefore, this study considered the addition of mixed beans to glutinous rice flour combined with HMT to reduce the eGI value of glutinous rice flour and sweet dumplings. By eliminating the off‐flavors of beans, we analyzed the effects of starch granule morphology, crystal structure, thermal properties, and protein secondary structure on the eGI value. Additionally, we observed the impacts of color, texture, coagulability, and odor on the quality of sweet dumplings. This research has provided a theoretical basis and technical guidance for developing low‐eGI foods suitable for patients with T2DM and other special populations.

## 2 METHODS

### 2.1 Preparation of mixed‐flour heat–moisture treatments and fast‐frozen sweet dumplings

**FIGURE 1:**
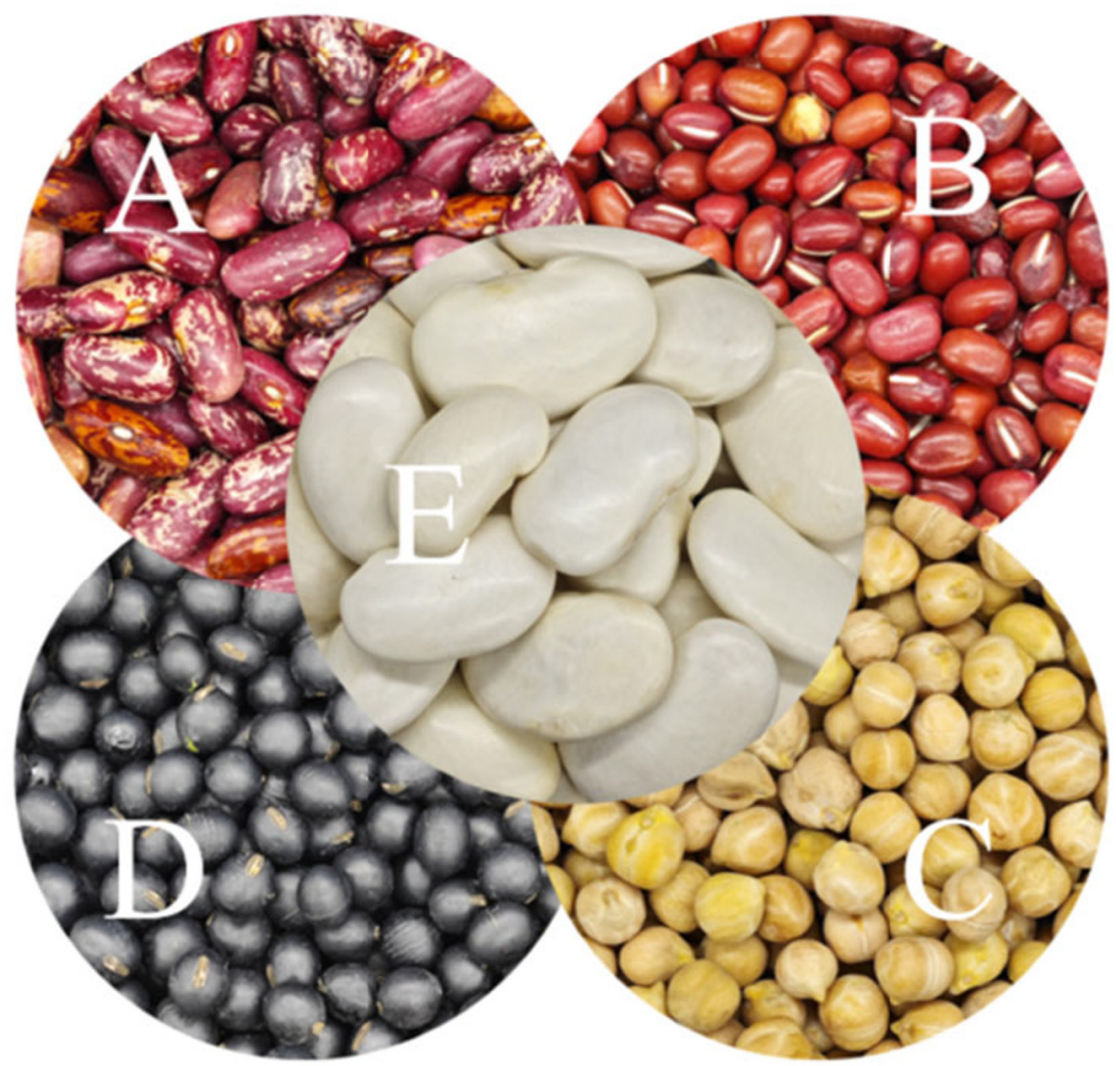
Pictures of various bean species (a, b, c, d, and e representing florid kidney bean, red adzuki bean, chickpea, black bean, and white kidney bean, respectively).

Li's research methods (Li et al., 2021) were referenced, and slight adjustments were made to their approach. The experimental materials were purchased from Bengbu Brothers Cereals and Oils Food Science and Technology Co., Ltd., Bengbu, China. For this experiment, we took 80 g of glutinous rice flour and 20 g of other bean flour (such as florid kidney bean, red adzuki bean, chickpea, black bean, and white kidney bean, as shown in Figure 1) and thoroughly mixed them. The moisture content of the blended powder was adjusted to 25% (Hoover & Vasanthan, 1994), loaded into a sealed jar (Metal sealing cans, Shanghai Liuyi Technology Ltd., China), and left to stand for 24 h. It was then processed in an oven at 110°C for 2 h to obtain the HMT mixed flour, and stored at 4°C. Hundred grams of prepared HMT mixed flour was combined with distilled water to form a dough. The dough had been wrapped with plastic wrap and allowed to rest for 20 min. The mixture was divided into 10 g portions and rolled into round shapes by hand to make fresh sweet dumplings. To facilitate quick freezing, the fresh dumplings were frozen for 30 min at −40°C. The sweet dumplings, which had been quickly frozen and individually wrapped in plastic wrap, were kept at −18°C. The final samples are displayed in Table 1.

**TABLE 1:** Mixture of glutinous rice with various beans, which synergized with HMT to produce the mixed flour and sweet dumplings.

Methods of treatment	Abbreviations
Glutinous rice flour	GRF
Glutinous rice flour with flower kidney beans added by heat–moisture treatments	F‐GRF
Glutinous rice flour with red adzuki bean added by heat–moisture treatments	R‐GRF
Glutinous rice flour with chickpea added by heat–moisture treatments	C‐GRF
Glutinous rice flour with black beans added by heat–moisture treatments	B‐GRF
Glutinous rice flour with white kidney beans added by heat–moisture treatments	W‐GRF
Sweet dumplings made with glutinous rice flour	G‐SD
Sweet dumplings made by mixing flower kidney beans with glutinous rice flour through heat–moisture treatments	F‐SD
Sweet dumplings made by mixing red adzuki bean with glutinous rice flour through heat–moisture treatments	R‐SD
Sweet dumplings made by mixing chickpea with glutinous rice flour through heat–moisture treatments	C‐SD
Sweet dumplings made by mixing black beans with glutinous rice flour through heat–moisture treatments	B‐SD
Sweet dumplings made by mixing white kidney beans with glutinous rice flour through heat–moisture treatments	W‐SD

### 2.2 Component analysis of HMT mixed flour

The fat, ash, total protein, and moisture content of the mixed flour described in Section 2.1 were determined using standard AOAC procedures. The nitrogen content was determined using an automatic Kieldahl apparatus (Kjel‐tec™ 8400, Foss Inc., Sweden), and the nitrogen conversion coefficient was set to 6.25.

### 2.3 Scanning electron microscope (SEM) of mixed flour

The prepared 2.1 mixed flour samples were spread out on a sample stage secured with conductive double‐sided tape and sprayed with platinum. In accordance with the research by Sun et al. (2014), the assay pressurization was adjusted to 20 kV. The shape and structure of the 2.1 mixed starch granules were examined using a scanning electron microscope (SEM) (TM3000, Hitachi Limited Co., Ltd., Tokyo, Japan) to magnify the images.

### 2.4 X‐ray diffraction (XRD) of mixed flour

The 2.1 mixed flour samples were placed in an XRD sample stage (X'Pert Pro, PANalytical, Almelo, The Netherlands) for testing in accordance with Sui et al. (2015) after being laid flat against the sample tank, compressed, and having excess sample removed. The diffraction test conditions were as follows: CuK α‐rays, tube voltage 36 kV, tube current 24 mA, scanning range 4 to 50°, scanning speed 2°/min, step size 0.02°. The x‐ray patterns obtained were analyzed using Jade 6.5 software to calculate the relative crystallinity (RS).

### 2.5 Fourier transform infrared spectroscopy (FTIR) of mixed flour

Using an FTIR spectrometer (Spectrum Two, PerkinElmer Co., Ltd., Massachusetts, MA, USA) and following the methodology of Li et al., (2020), the 2.1 mixed flour samples were combined in a 1:100 ratio with potassium bromide. A suitable amount of the mixed sample was taken and pressed into a complete transparent sheet of 1 mm thickness, then scanned in the wave number range of 400–4000 cm^−1^ with a resolution of 4 cm^−1^. The scanning time was 16 s, and the number of scans was 32.

### 2.6 Mixolab analysis for mixed flour

The thermo‐mechanical special energy of the 2.1 mixed flour was examined using a Mixolab analyzer (MIXOLAB 2, France Chopin Technology Co., Ltd., Paris, France). The methods used in this study were based on those of Wang and Copeland (2013). The mixed flour was blended with distilled water to prepare 75 g of dough. Dual mixing was conducted at 80 rpm, and the temperature of the water tank was maintained at 30°C. The parameters were then recorded.

### 2.7 RVA analysis for mixed flour

According to Mudgil et al. (2016), the adhesion properties of mixed flour in 2.1 were evaluated using the Rapid Viscosity Analyzer (RVA) (RVA‐3D; Newport Scientific, Narrabeen, Australia). A total of 3.5 g of mixed flour from 2.1 (14% wet basis) was mixed with 25 mL of distilled water in an aluminum cylinder, which was rotated five times using a plastic stirring paddle before insertion into the instrument. The temperature ramping protocol for the RVA test was as follows: the sample was held at 50°C for 1 min, then uniformly heated to 95°C at a rate of 12°C/min, maintained at 95°C for 2.5 min, and finally uniformly cooled to 50°C at a rate of 12°C/min, where it was held for 2 min.

### 2.8 DSC analysis for mixed flour

The thermal properties of starch in the 2.1 mixed flour were assessed using differential scanning calorimetry (DSC) (DISCOVERY DSC 250, Waters Technology (Shanghai) Ltd., China), following the refined method outlined in the study by Perera et al. (1997). A 3 mg sample was weighed into a 40 μL aluminum crucible, followed by the addition of 9 μL of distilled water for thorough mixing. The crucible was then sealed and allowed to equilibrate at room temperature for 24 h. Using a blank crucible as a reference, the differential scanning calorimeter measured the sample over a temperature range of 25°C to 125°C at a heating rate of 10°C/min. Parameters such as the initial pasting temperature (To), peak pasting temperature (Tp), termination pasting temperature (Tc), and enthalpy of pasting (∆H) were recorded for the samples.

### 2.9 In vitro starch digestion assay of mixed flour and fast‐frozen sweet dumplings

The eGI values and in vitro digestive properties of mixed starches were determined using slightly modified versions of the techniques from Pautong et al. (2022) and Englyst et al. (1999). About 200 mg of 2.1 mixed flour was weighed into a 100‐mL centrifuge tube. Then, 20 mL of 0.3 mol/L NaCl (0.6 mol/L HCl adjusted to pH = 2) was added, and the mixture was pasted in a boiling water bath for 30 min. After cooling to room temperature, 30 mL of phosphate buffer solution was added. Then, 1 mL of 150 U/mL glucosidase (Hefei Qiansheng Bio‐Tech Ltd., China) and 4 mL of 725 U/mL pancreatic α‐amylase (Hefei Qiansheng Bio‐Tech Ltd., China) were added, and the mixture was digested by shaking in a water bath shaker at 40°C, 150 r/min. Subsequently, at 0, 10, 20, 30, 60, 90, 120, and 180 min, 1 mL of the supernatant was transferred into a 1.5 mL centrifuge tube and boiled in a water bath for 5 min. Finally, the supernatant was used as the sample solution, and a glucose kit (Glucose Assay Kit, Nanjing Jiancheng Bioengineering Institute, China) was employed to determine the glucose content. The content of Rapidly Digestible Starch (RDS), Slowly Digestible Starch (SDS), and Resistant Starch (RS) was calculated using the following formula:

(1) $$\mathrm{RDS}|\%=\frac{G20-G0}{\mathrm{TS}}\times 0.9\times 100$$

(2) $$\mathrm{SDS}|\%=\frac{G120-G20}{\mathrm{TS}}\times 0.9\times 100$$

(3) $$\mathrm{RS}|\%=\frac{\mathrm{TS}-\mathrm{RDS}-\mathrm{SDS}}{\mathrm{TS}}\times 0.9\times 100$$

where G0, G20, and G120 represent the glucose content at 0, 20, and 120 min, respectively, and TS is the total starch content. The corresponding curves were plotted with oscillation time as the horizontal axis and the rate of starch hydrolysis as the vertical axis. The Hydrolysis Index (HI) was defined as the ratio of the area under the hydrolysis curve of the sample to that of the white bread. The eGI was calculated using the following formula.

(4) eGI=0.862HI+8.198

The five frozen sweet dumplings prepared in step 2.1 were placed into a 500‐mL beaker of distilled water and boiled for 6 min, then removed and drained. Subsequently, they were boiled for another 6 min, followed by removal and draining. The moisture content was reduced to less than 14% in an oven at 40°C. The material was then pulverized and sieved (80 mesh). The digestive properties and estimated Glycemic Index (eGI) values of the sweet dumplings were determined through simulated in vitro digestion.

### 2.10 Color measurement of fast‐frozen sweet dumplings

The color coordinates of the 2.1 fast‐frozen sweet dumplings were measured at room temperature using a CIE Lab class colorimeter (Hunterlab, Miniscan EZ, USA): L brightness, a* chromaticity, and b* chromaticity. In this experiment, six sets of samples were measured six times each, and the values of L*, a*, and b* were recorded. The total color difference (ΔE) was calculated according to the equations proposed by Duangmal et al. (2008).
(5)

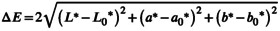




L_0_* = 99.50, a_0_* = − 0.06, and b_0_* = − 0.19 were the color parameters of the white standard plate.

### 2.11 E‐nose analysis of fast‐frozen sweet dumplings

The odor of quick‐frozen dumplings before and after cooking was determined using an electronic nose (AIRSENSE Electronic Nose PEN 3, Ensoul Technology Co., Ltd., Beijing, China). The study referenced Guohua et al. (2012) as its basis, where a model was developed and the experimental data were analyzed using Principal Component Analysis (PCA) and Linear Discriminant Analysis (LDA).

### 2.12 TPA analysis for fast‐frozen sweet dumplings

After the quick freezing of the sweet dumplings from 2.1, they were cooked and subjected to texture profile analysis (TPA) using a CT‐3 instrument (Brookfield Co., Ltd., MA, USA). Hardness, adhesiveness, viscoelasticity, and chewiness were analyzed according to the method described by Rahman and Al‐Farsi (2005). The TPA parameters were set as follows: TPA mode; TA4 probe model; pre‐test rate of 2 mm/s; mid‐test rate of 1 mm/s; post‐test rate of 1 mm/s; compression ratio of 50%; and two compression dwell times of 5 s. The compression rate was set at 1 mm/s.

### 2.13 Transmittance of the soup and loss of fast‐frozen dumplings after boiling

According to the method of a previous study (Zhang et al., 2021), five sweet dumplings were added to 500 mL of distilled water and boiled for 6 min. After cooling to room temperature, the broth was transferred into a 500‐mL volumetric flask and adjusted to 500 mL. Transmission at 620 nm was measured using distilled water as a control. The soup was collected and evaporated to dryness, and the mass of the remaining solids was calculated as the loss.

### 2.14 Cracking rate and water loss of fast‐frozen sweet dumplings

Fast‐frozen sweet dumplings were categorized as cracked or uncracked. If they exhibited visible cracks on the surface, they were recorded as 0; otherwise, they were recorded as 1. During freezing and thawing, the weight of fast‐frozen sweet dumplings decreased due to moisture loss, so the difference in weight before and after the experiment represented the amount of moisture loss (Li et al., 2018).

### 2.15 Statistical analysis

The results were expressed as the mean ± standard deviation of three replicate experiments. Data were analyzed using one‐way analysis of variance (ANOVA), followed by Duncan's multiple range test using the SPSS 17.0 statistical software program (SPSS Inc., Chicago). *p* < .05 was considered statistically significant.

## 3 RESULTS AND DISCUSSION

### 3.1 Proximate composition of mixed flour

The proximate composition of the glutinous rice mixture, after the addition of other beans and synergistic HMT, is shown in Table 2. The contents of various components in all mixed flours were increased to varying degrees, with significant increases observed in ash and protein content. This indicates that legumes are rich in mineral salts and high‐quality protein components. The abundant protein components in legumes effectively inhibited the entry of amylase, providing a basis for reducing the eGI value of glutinous rice mixtures. Under conditions of high temperature and high moisture, the starch double helix structure was opened and combined with lipids and proteins. This had resulted in the formation of protein–starch–lipid complexes, as described by Dias et al. (2010). The material facilitated the reorganization of starch molecules into a dense configuration, which hindered the penetration of amylase into the interior of the starch molecules and decelerated the hydrolysis rate of a portion of the starch (Khunae et al., 2007).

**TABLE 2:** Approximate composition of the HMT composite mixed flour.

Samples	Ash/%	Lipid/%	Moisture content/%	Protein/%
GRF	0.225 ± 0.01^e^	10.9 ± 0.35^bc^	11.62 ± 0.07^b^	6.88 ± 0.02^e^
F‐GRF	0.584 ± 0.01^c^	10.57 ± 0.12^c^	11.91 ± 0.03^a^	10.51 ± 0.01^bc^
R‐GRF	0.485 ± 0.05^d^	10.89 ± 0.31^bc^	11.87 ± 0.03^a^	10.92 ± 0.05^b^
C‐GRF	0.503 ± 0.01^d^	11.51 ± 0.82^b^	11.85 ± 0.09^a^	9.83 ± 0.11^c^
B‐GRF	0.65 ± 0.01^a^	15.88 ± 0.41^a^	11.26 ± 0.14^c^	15.28 ± 0.06^a^
W‐GRF	0.615 ± 0.02^b^	11.03 ± 0.44^bc^	12.05 ± 0.03^a^	9.96 ± 0.12^c^

*Note*: Assays were performed in triplicate. Mean ± SD values in the same column with different superscript letters are significantly different (*p* < .05).

### 3.2 Observation on the morphology and structure of starch granules in mixed flour

**FIGURE 2:**
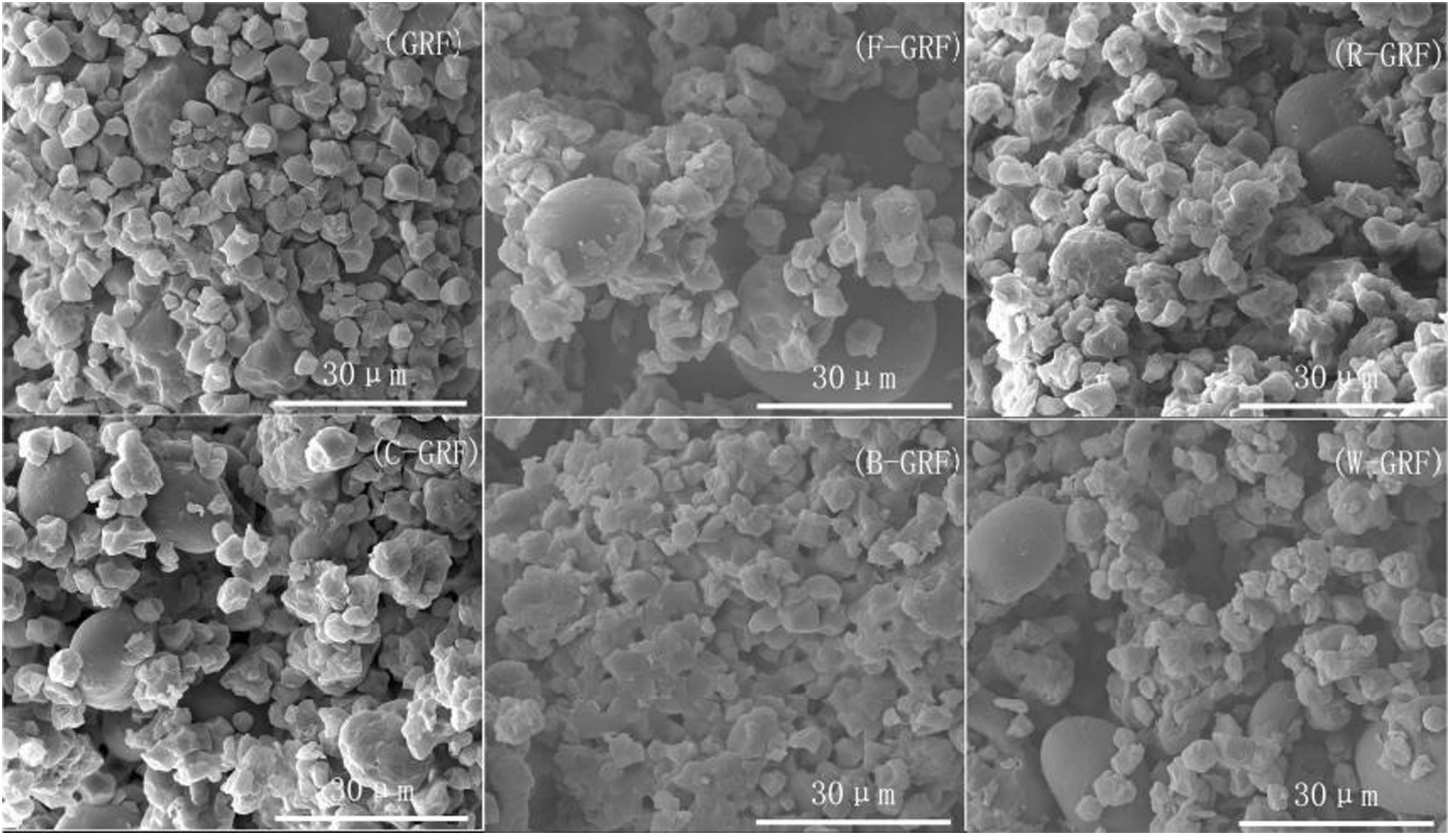
SEM image of HMT mixed flour.

The starch granule morphology of HMT mixed flour is shown in Figure 2. The illustration clearly demonstrates a noticeable discrepancy in the surface microstructure of the combined flour. The surface morphology of the processed mixed flour showed a denser structure, with a notable decrease in the spacing between starch granules and a significant increase in agglomeration, thereby impeding amylase penetration. The reason for the decrease in eGI value of the mixed flour had been further verified, supported by the research results of Gonzalez and Wang (2023). The R‐GRF starch granules exhibited minimal morphological disruption, maintaining an overall structure that remained largely intact. They significantly minimized the gaps between the starch granules, resulting in a high level of integrity. The more solid structure of the red kidney bean starch granules, which successfully prevented HMT‐dextrinization, could explain this. Additionally, the protein network structure better encapsulated the starch flour granules. The reduced interaction between starch granules and enzymes, along with decreased sensitivity to amylase, reduced the rate of hydrolysis of mixed starches and decreased their eGI values (H. Wang et al., 2017).

### 3.3 Thermo‐mechanical characterization of mixed flour

The torque produced while the dough was kneaded by the two arms was recorded by the Mixolab apparatus in order to analyze the quality of gluten and the behavior of starch pasting. The thermomechanical properties of the mixed flour have been measured, and the results are shown in Table 3. The parameters C2, C3, and C4 were used to measure the extent of starch pasting in the dough, enzyme activity, and the qualities of the dough (Xu et al., 2019). Understanding the degree of dough pasting was critical for assessing starch digestibility. In this regard, C2 served as a significant indicator for assessing both starch digestibility and the extent of starch pasting. The torque of the HMT blend was much higher compared to GRF C2 (0.209 N/m), suggesting that it was more difficult to stick together compared to the GRF. This observation indicated that the reduced enzymatic degradation of the blended starch could be attributed to this difference (Jia et al., 2011). This outcome aligned with investigations into the amylase activity of C3‐C4. By analyzing the C3‐C2 data, it was found that the addition of other legumes to GRF, synergistically with the HMT method, effectively reduced the degree of pasting and increased the agglomeration of the mixed flour. It was found that this effectively hindered amylase digestion and reduced starch digestibility, and was also essential for improving dough quality and enhancing the quality of sweet dumplings. Table 3 shows the correlation between C4/C3 and cooking performance. It was found that, compared to GRF, the cooking performance of the mixed flour was significantly improved after treatment. The study demonstrated that sweet dumplings made from mixed flour experienced less exudation of moisture and starch during the cooking process, which contributed to the improved cooking quality of the sweet dumplings (Patel & Seetharaman, 2010).

**TABLE 3:** Thermomechanical properties, thermodynamics, and secondary protein structure of the HMT mixed flour.

Samples	GRF	F‐GRF	R‐GRF	C‐GRF	B‐GRF	W‐GRF
C2/N·m	0.209 ± 0.00^e^	0.285 ± 0.00^c^	0.266 ± 0.10^d^	0.308 ± 0.00^b^	0.597 ± 0.01^a^	0.271 ± 0.01^c^
C3/N·m	0.637 ± 0.10^b^	0.515 ± 0.01^c^	0.512 ± 0.00^c^	0.515 ± 0.03^c^	0.901 ± 0.06^a^	0.528 ± 0.12^c^
C4/N·m	0.420 ± 0.00^b^	0.348 ± 0.00^d^	0.367 ± 0.04^c^	0.352 ± 0.02^cd^	0.757 ± 0.00^a^	0.37 ± 0.00^c^
C3‐C2/N·m	0.428 ± 0.00^b^	0.23 ± 0.03^d^	0.246 ± 0.05^cd^	0.207 ± 0.00^d^	0.304 ± 0.00^c^	0.258 ± 0.00^cd^
C3‐C4/N·m	0.217 ± 0.00^a^	0.167 ± 0.00^b^	0.145 ± 0.00^d^	0.163 ± 0.01^b^	0.144 ± 0.01^cd^	0.158 ± 0.03^c^
C4/C3/N·m	0.659 ± 0.01^c^	0.675 ± 0.10^c^	0.716 ± 0.00^b^	0.683 ± 0.00^c^	0.840 ± 0.02^a^	0.701 ± 0.11^b^
To(°C)	50.91 ± 0.02^c^	51.18 ± 0.02^c^	69.27 ± 0.05^a^	35.63 ± 0.04^e^	44.16 ± 0.05^d^	60.66 ± 0.01^b^
Tp(°C)	92.27 ± 0.04^d^	99.40 ± 0.04^c^	105.75 ± 0.02^a^	104.06 ± 0.06^ab^	100.44 ± 0.06^bc^	100.02 ± 0.05^c^
Tc(°C)	121.73 ± 0.05^ab^	116.38 ± 0.02^c^	123.14 ± 0.02^a^	121.72 ± 0.02^ab^	118.34 ± 0.06^bc^	125.16 ± 0.01^a^
ΔHg(J/g)	987.54 ± 3.50^e^	1493.10 ± 2.09^c^	1637.91 ± 1.06^b^	1275.61 ± 3.28^d^	1497.61 ± 5.88^c^	1525.55 ± 4.11^c^
β‐Sheet/%	43.41 ± 0.00^a^	37.32 ± 0.01^c^	33.88 ± 0.00^e^	36.06 ± 0.05^d^	37.70 ± 0.13^d^	39.31 ± 0.12^b^
α‐Helix/%	16.95 ± 0.01^e^	17.49 ± 0.04^b^	19.00 ± 0.00^c^	18.05 ± 0.07^a^	18.11 ± 0.03b^c^	17.93 ± 0.07^d^
β‐Turn/%	21.28 ± 0.01^d^	27.18 ± 0.03^ab^	28.61 ± 0.14^b^	26.05 ± 0.5^ab^	24.78 ± 0.17^a^	22.39 ± 0.92^c^
Random coil/%	18.34 ± 0.21^c^	17.99 ± 0.03^c^	18.49 ± 0.87^c^	19.82 ± 0.11^a^	19.40 ± 0.05^bc^	20.35 ± 0.02^ab^

*Note*: Assays were performed in triplicate. Mean ± SD values in the same column with different superscript letters are significantly different (*p* < .05).

### 3.4 Thermodynamic characterization of mixed flour

The results of Table 3 show that the thermodynamic properties of starch samples could be assessed using four parameters: onset temperature (To), peak temperature (Tp), termination temperature (Tc), and enthalpy of pasting (Hg). Significant differences in the mixed flour's (To), (Tp), (Tc), and (ΔHg) were observed after undergoing HMT. The enthalpy of gelatinization (ΔHg) was positively correlated with crystallinity, and the value of ΔHg significantly increased, leading to an increase in the energy of the melted crystals. By examining Table 3, it was observed that the addition of red adzuki beans enhanced the crystallinity of R‐GRF starch. This substance exhibited excellent stability and strong resistance to amylase, resulting in a slower rate of starch hydrolysis and a lower in vitro digestion eGI value. This finding had been corroborated by the research conducted by Lewandowicz et al. (2000) and had been consistent with X‐diffraction observations.

Straight‐chain starch contained multiple α‐1,4 glycosidic linkages, which were rigid and required higher temperatures to form a paste. Consequently, as shown in Table 3, the content of straight‐chain starch has been positively correlated with the paste onset temperature (Chung et al., 2003). Consequently, the initial pasting temperature of R‐GRF was higher compared to other samples, indicating a higher concentration of straight‐chain starch in R‐GRF. On the other hand, amylase had struggled to catalyze enzymatic reactions with straight‐chain starch. This might have been the reason why the R‐GRF sample had the lowest eGI value during in vitro digestion, as explained by the research of Chen, He, Fu, et al. (2017). Due to the addition of red adzuki beans, the amount of amylose in the R‐GRF samples increased, leading to a change in the ratio of amylose to amylopectin. Under HMT conditions, amylose had readily reacted with lipids to form starch–lipid complexes, which had enriched the protein network structure and enhanced the interactions between starch granules, thereby increasing starch stability (Chen et al., 2015; Silva et al., 2017). This substance enhanced the enzyme resistance of the starch, resulting in a lower eGI effect.

### 3.5 Paste characterization of mixed flour

**FIGURE 3:**
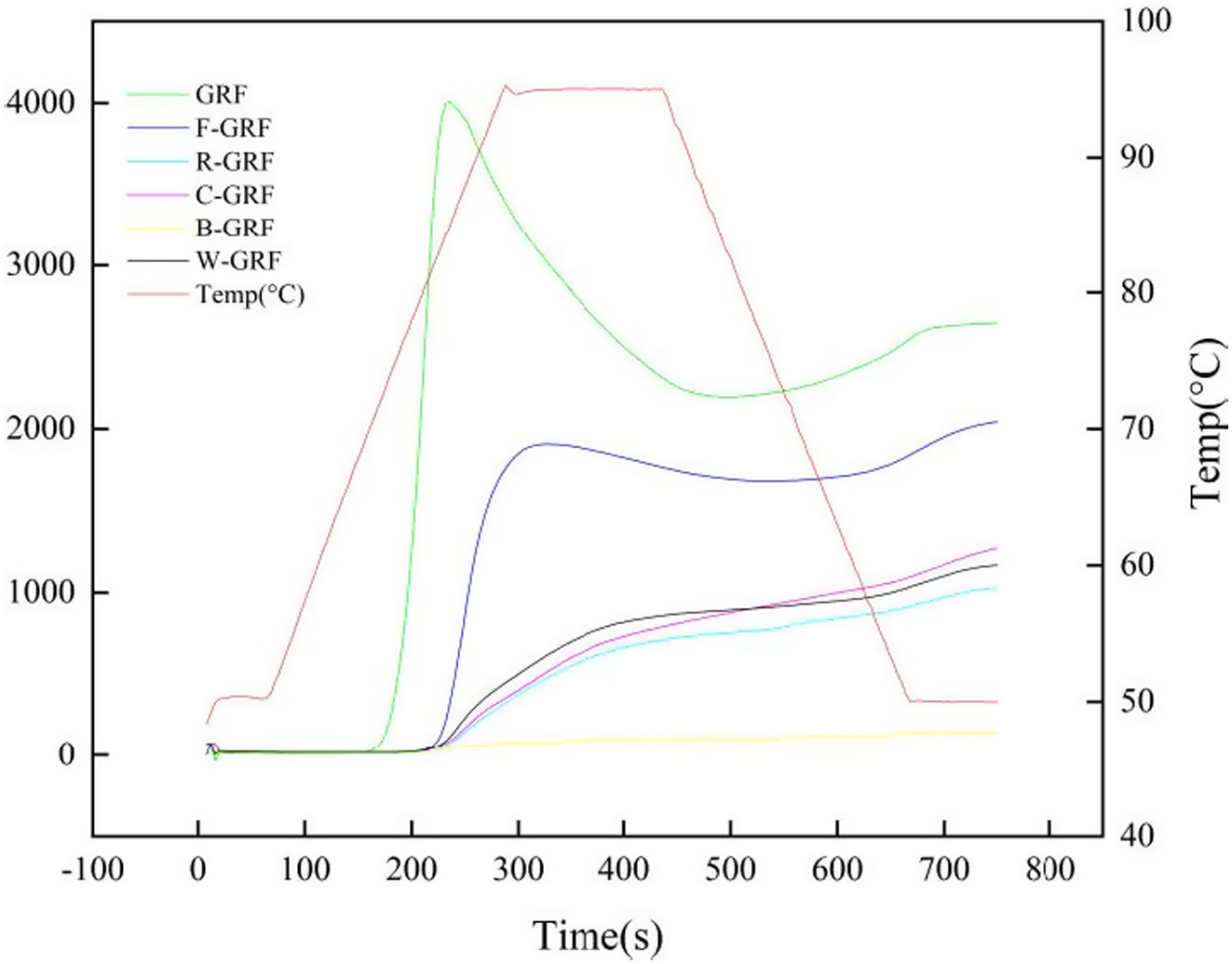
RVA profile analysis of HMT mixed flour.

The viscosity profiles of glutinous rice flour and HMT‐blend glutinous rice flour are shown in Figure 3. The addition of legumes to glutinous rice flour, combined with the dual method of HMT, was found to significantly reduce the viscosity properties of the mixed flour. This reduction may be related to the reorganization of starch molecular chains, which promotes the tight alignment of double‐helical structures. Additionally, the increased ratio of amylose to amylopectin enhanced the rigidity of the structure, thereby restricting starch swelling and reducing viscosity (Jiranuntakul et al., 2011). Another possibility was that the high temperature and high moisture content of the environment caused the rich proteins in the beans and the starch in the glutinous rice flour to combine, forming a protein–starch complex. This complex effectively inhibited the swelling of the starch, thereby lowering the viscosity (Puncha‐arnon & Uttapap, 2013). Compared to the other samples, the peak viscosity curve of B‐GRF showed less variation. This was speculated to be because black beans contained significantly more protein and fat than the other samples. Some of these proteins and fats produced ternary comonomers (protein–starch–fat complexes) with starch under HMT conditions, limiting starch swelling (Su et al., 2020). Second, the substantial quantity of unbound proteins and lipids created a physical barrier that restricted starch swelling and further lowered viscosity. Ragaee and Abdel‐Aal (2006) found that heat treatment denatured proteins. This increased the interaction force between starch and denatured proteins, which slowed the swelling of starch granules and reduced their viscosity.

### 3.6 Protein secondary structure of mixed flour

The secondary structure of glutelin was quantified using peak fit fitting software by fitting the second derivative of Fourier transform infrared spectroscopy. Protein secondary structures, commonly observed as β‐sheet, α‐helix, and β‐turn, are summarized in Table 3. Protein secondary structure analysis in mixed flours revealed a significant increase in α‐helix and β‐turn and a notable decrease in β‐sheet. R‐GRF had reduced β‐sheet content by 21.95% and had increased β‐turn and α‐helix content by 25.62% and 11.05%, respectively, compared to GRF. Under high temperature and humidity conditions, the unfolding and recombination of protein β‐sheet peptide chains and starch double‐helix structures may have caused the observed decrease in β‐sheet content. This recombination process then initiated a cross‐linking reaction, ultimately producing a protein–starch complex. The material's ability to effectively reduce the rate of enzymatic starch breakdown and slow the glycemic index effect might have been one of the reasons for the low eGI value of R‐GRF (Bock & Damodaran, 2013).

### 3.7 Starch crystal structure analysis of mixed flour

**FIGURE 4:**
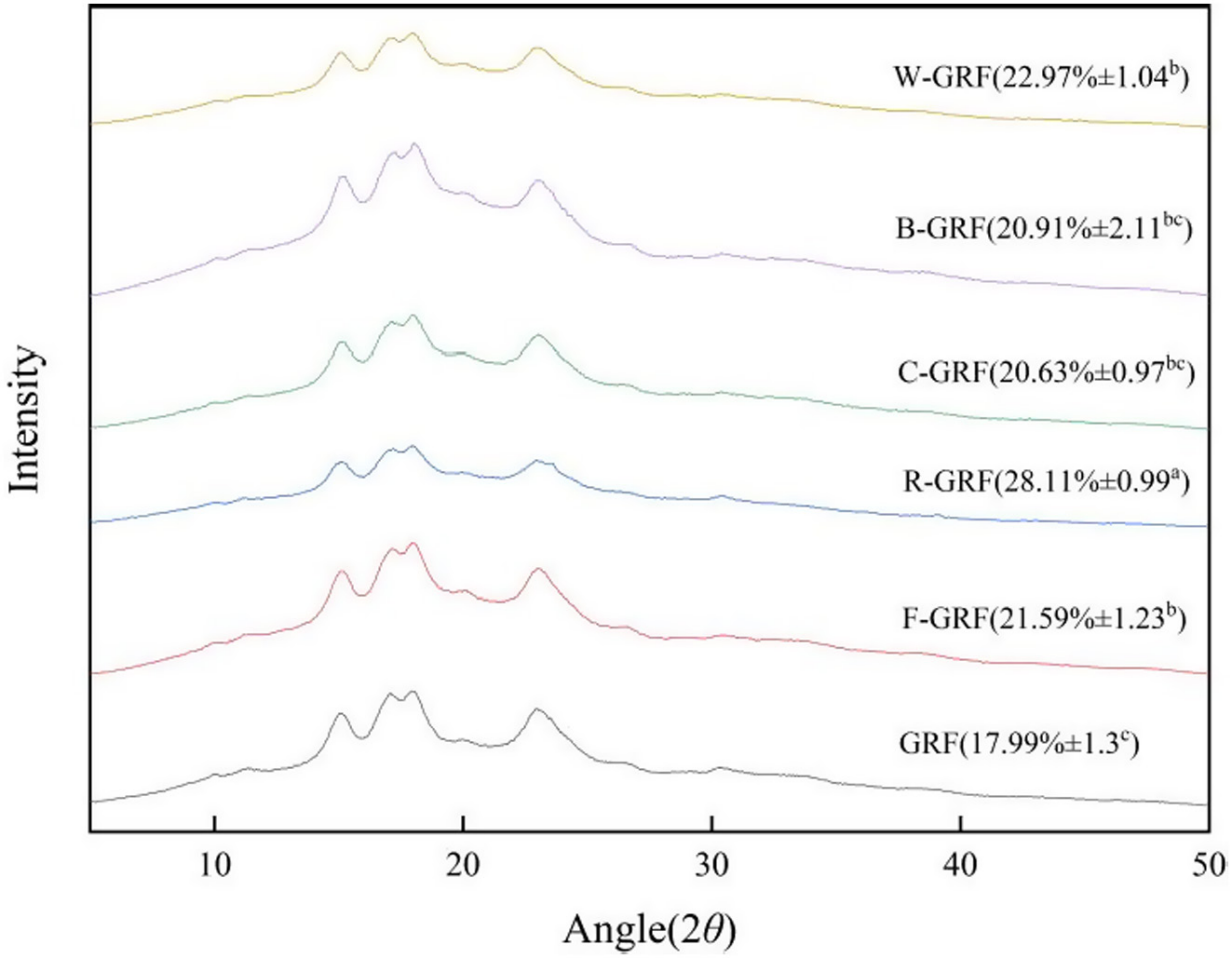
XRD pattern analysis of HMT mixed flour.

The crystal structure of the mixed glutinous rice flour had been identified as a typical A‐type when the characteristic peaks of the X‐ray diffraction corresponded to 15°, 17°, 18°, and 23° (Blazek & Gilbert, 2011). All of the glutinous rice mixed flour crystallinity had increased significantly under HMT settings, as shown in Figure 4, with the R‐GRF crystallinity of 28.11 ± 0.99% having risen by 36% in comparison to the GRF crystallinity of 17.99 ± 1.3%. The study found that combining beans with HMT could greatly improve the crystallinity of glutinous rice flour. This improvement might have occurred under high temperature and humidity conditions, where the internal structure of starch granules cross‐linked with proteins, resulting in protein–starch complexes (Wang et al., 2020). These complexes had demonstrated an enhanced ability to stimulate recrystallization within the starch granules, thereby increasing the relative crystallinity of the starch. The current experimental results were aligned with the findings from DSC and FTIR analyses.

### 3.8 Comparison of the in vitro digestive properties of starch from mixed flour and fast‐frozen sweet dumplings

The starch digestion characteristics and eGI values of HMT mixed flour and sweet dumplings are listed in Table 4. The addition of legumes combined with the HMT method significantly reduced the digestibility of mixed flour and sweet dumpling starch. The RDS content had significantly decreased, and the RS content had increased, resulting in a decrease in the eGI values. When comparing GRF to R‐GRF, the RS content in R‐GRF had increased to 55.54%, while the eGI had decreased to 52.01%, achieving the expected effect of lowering eGI values. The results of this study aligned with Cappa et al. (2020) findings, which discovered a negative correlation between RDS content and legume addition, and a positive correlation between RS content and legume addition. According to Lal et al. (2021) studies, as RS content increased, the starch hydrolysis rate and glucose release rate decreased, leading to a reduction in the glycemic index (GI). Beans are rich in proteins, lipids, and other nutrients; red adzuki beans, in particular, are high in flavonoids and polyphenols, which have a significant inhibitory effect on amylase hydrolysis (Chen et al., 2017). HMT mixed flour had been able to form protein–lipid–starch complexes, which could enhance the compact arrangement of starch molecules in a double‐helix structure and adhere to the surface of starch granules. According to Chen et al. (2018), this material had successfully prevented amylase from penetrating the starch interior and had reduced its interaction sites, thereby lowering the rate of starch hydrolysis and positively impacting the reduction of eGI values. The digestion results of the sweet dumpling starch had been consistent with those of the mixed flour starch.

**TABLE 4:** Starch digestion properties and eGI values of the mixed flour and sweet dumplings.

Samples	RDS/%	SDS/%	RS/%	eGI
GRF	44.38 ± 0.34^a^	24.01 ± 0.23^b^	31.58 ± 0.58^e^	82.96 ± 1.11^a^
F‐GRF	29.73 ± 0.29^c^	21.49 ± 0.52^c^	48.26 ± 0.32^c^	60.79 ± 0.59^d^
R‐GRF	25.42 ± 0.44^d^	19.01 ± 0.67^f^	55.54 ± 0.23^a^	52.01 ± 1.41^f^
C‐GRF	26.14 ± 0.39^d^	21.65 ± 0.44^d^	52.69 ± 0.33^b^	56.12 ± 1.01^e^
B‐GRF	32.52 ± 0.62^b^	24.13 ± 0.59^a^	42.84 ± 0.72^d^	69.39 ± 0.95^b^
W‐GRF	31.67 ± 0.75^b^	21.12 ± 0.47^e^	47.68 ± 0.72^c^	63.28 ± 1.87^c^
G‐SD	32.24 ± 0.65^ab^	17.10 ± 0.13^b^	50.65 ± 0.25^cd^	61.60 ± 2.88^ab^
F‐SD	27.98 ± 0.38^cd^	19.49 ± 0.23^a^	52.51 ± 0.73^c^	56.47 ± 1.07^c^
R‐SD	25.56 ± 0.29^d^	18.57 ± 0.98^ab^	55.85 ± 0.15^b^	52.21 ± 1.44^d^
C‐SD	27.49 ± 0.10^c^	15.82 ± 0.51^c^	56.67 ± 0.55^b^	54.95 ± 0.51^cd^
B‐SD	33.05 ± 0.22^a^	18.50 ± 0.71^ab^	48.43 ± 0.22^d^	63.56 ± 1.95^a^
W‐GRF	29.47 ± 0.36^bc^	18.71 ± 0.58^b^	51.81 ± 0.18^c^	59.03 ± 1.37^b^

*Note*: Assays were performed in triplicate. Mean ± SD values in the same column with different superscript letters are significantly different (*p* < .05).

The study by Huang et al. (2016) demonstrated that HTM conditions had altered the ratio of RDS, SDS, and RS, encouraged the tight arrangement of starch molecules, and modified the starch structure. Significant changes in RDS, SDS, and RS were observed in mixed flour and sweet dumplings (Table 4). The potential cause of these substantial variations was linked to the alteration in starch granule structure and the increase in intermolecular forces. It was also possible that the starch molecular chains had undergone rearrangement and reorientation. This resulted in the entanglement of the branched terminal molecular chains within the starch granules, thereby increasing the interaction forces between the granules. The findings observed through SEM analysis supported this hypothesis (Gunaratne, 2002). The RS content in B‐GRF had been higher than in GRF, likely because the higher free fat content in black beans had impeded hydrolysis by amylase and increased starch resistance, consistent with the findings of De La Hera et al. (2014). Interestingly, the RS content in B‐GRF had been lower than in other treatment groups, possibly because the fat had readily undergone oxidation and decomposition under high temperatures, generating significant heat energy. This heat energy may have caused more severe damage to the shape and structure of starch granules. The broken starch granules had reduced the entanglement of branched starch molecular chains, weakened the straight‐chain–branched‐chain interaction force, and thus decreased the stability of the starch molecular structure (Kunyanee & Luangsakul, 2022). Additionally, the higher eGI value in B‐SD may have been due to the further breakdown of starch structure during dumpling processing, which had weakened the starch resistance.

### 3.9 Comparison of the quality characteristics of fast‐frozen sweet dumplings

**FIGURE 5:**
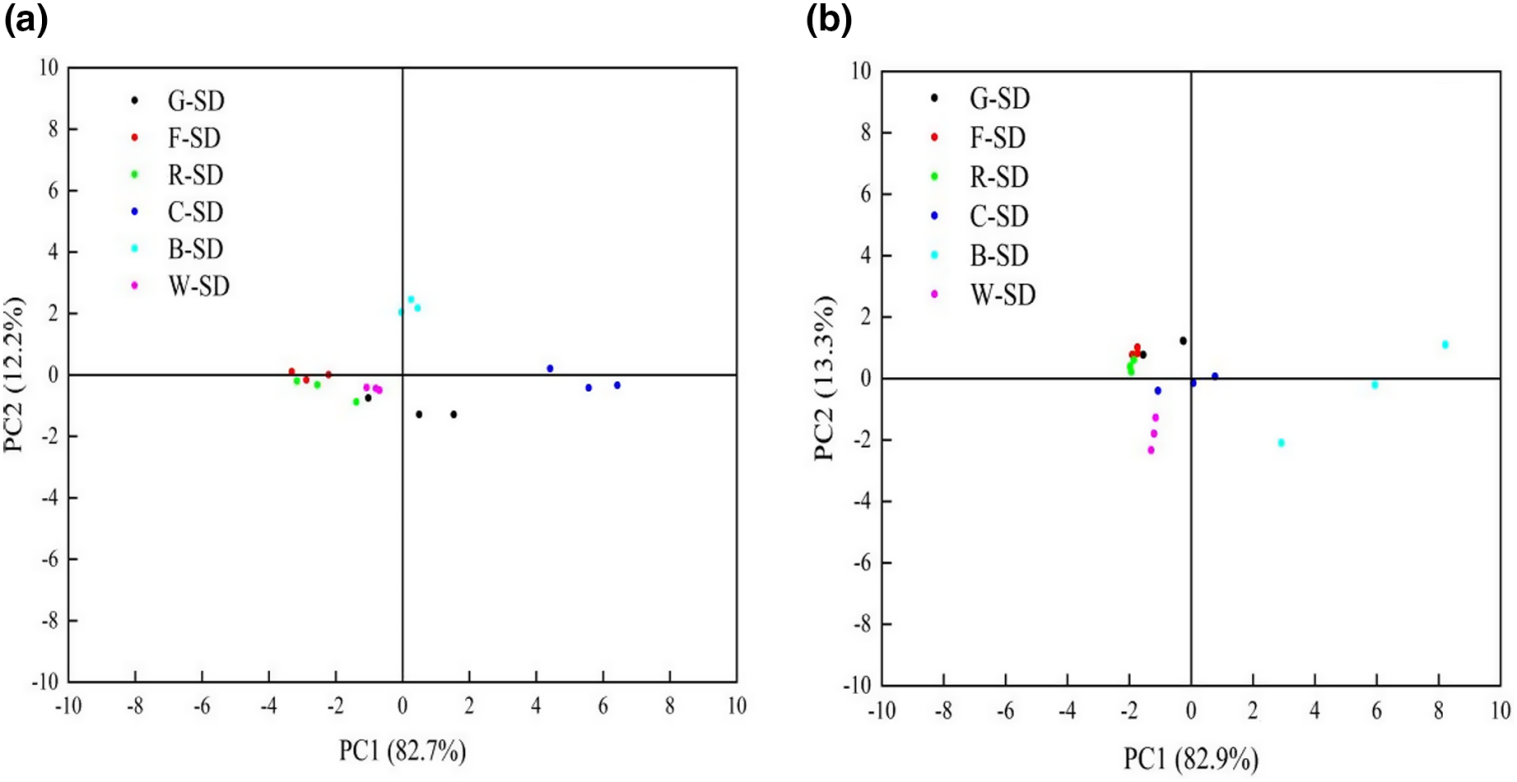
PCA of the e‐nose data for sweet dumplings before and after cooking. Sweet dumplings before steaming (a); sweet dumplings after steaming (b).

Several key criteria, such as texture, quick‐freezing index, characteristics, color, and odor, were used to assess the quality of sweet dumplings. These parameters are further described in Table 5 and Figure 5. The findings demonstrated substantial alterations in textural characteristics, including firmness, viscoelastic behavior, and chewiness. Hardness, often regarded as the most crucial metric for texture analysis, had been positively correlated with the chewiness metric, which is a product of viscosity and elasticity. Interestingly, in contrast to the findings of Rahman & Al‐Farsi, (2005), the inclusion of red adzuki bean reduced the sweet dumplings' hardness while increasing their chewiness. The addition of red adzuki beans likely made the R‐GRF dumplings stickier, increasing the resistance during chewing and thereby enhancing the chewiness of R‐SD. From the analysis of quick‐freezing quality and overall quality, it was shown that the addition of other beans to glutinous rice flour significantly reduced the cracking rate and water loss of sweet dumplings and improved their transmittance through the dual action of HMT. This could have been due to the high protein and fat content in the beans, which increased adhesiveness and reduced the exudation of water and starch. The enhanced density of the double‐helix configuration had facilitated the close structure between starch granules, thereby improving the water retention and gelatinous properties of the sweet dumplings, ultimately enhancing their overall quality. These results aligned with the findings of thermomechanical analysis trials.

**TABLE 5:** Texture parameters, quality attributes, and color of the sweet dumplings.

Samples	G‐SD	F‐SD	R‐SD	C‐SD	B‐SD	W‐SD
Hardeness/g	1138.04 ± 3.52^a^	558.21 ± 2.31^d^	356.33 ± 3.19^e^	1017.18 ± 4.85^b^	1030.36 ± 1.56^b^	640.52 ± 2.25^c^
Viscidity/mJ	0.99 ± 0.01^de^	1.51 ± 0.31^ab^	1.72 ± 0.12^a^	1.31 ± 0.21^bc^	1.13 ± 0.01c^d^	0.71 ± 0.01^e^
Springiness/mm	10.22 ± 1.12^a^	8.77 ± 0.52^a^	8.53 ± 0.51^a^	9.11 ± 0.09^a^	8.4 ± 0.38^a^	9.04 ± 1.21^a^
Chewiness/mJ	101.81 ± 2.19^c^	30.52 ± 1.88^e^	174.39 ± 3.35^b^	51.48 ± 0.99^d^	46.91 ± 1.51^de^	34.02 ± 1.33^de^
Cracking rate/%	64.33 ± 1.71^a^	58.01 ± 2.11^b^	33.19 ± 0.79^e^	51.23 ± 1.01^c^	29.08 ± 1.26^f^	45.81 ± 2.33^d^
Water loss/%	1.82 ± 0.03^a^	1.48 ± 0.05^b^	0.93 ± 0.06^de^	1.41 ± 0.02^bc^	0.69 ± 0.05^e^	1.15 ± 0.08^cd^
Cooking loss/%	6.69 ± 0.96^a^	5.83 ± 0.88^b^	4.95 ± 0.23^d^	5.52 ± 0.61^bc^	4.71 ± 0.33^d^	5.33 ± 0.13^c^
Transmittance/%	71.22 ± 2.21^e^	78.51 ± 1.22^c^	89.09 ± 0.85^b^	82.01 ± 1.55^cd^	93.33 ± 0.71^a^	84.34 ± 2.07^c^
L*	99.99 ± 0.66^a^	95.52 ± 0.78^b^	94.08 ± 0.67^c^	99.98 ± 0.64^a^	89.64 ± 0.68^d^	99.98 ± 0.76^a^
a*	0.38 ± 0.01^e^	10.31 ± 0.02^a^	8.43 ± 0.01^c^	4.76 ± 0.01^d^	9.08 ± 0.03^b^	4.88 ± 0.02^d^
b*	4.98 ± 0.03^d^	2.19 ± 0.01^e^	8.79 ± 0.02^c^	15.42 ± 0.02^b^	17.93 ± 0.03^a^	15.12 ± 0.01^b^
ΔE	0.00 ± 0.00^e^	11.28 ± 0.03^b^	10.68 ± 0.03^c^	11.32 ± 0.01^b^	18.69 ± 0.01^a^	11.09 ± 0.02^b^

*Note*: Assays were performed in triplicate. Mean ± SD values in the same column with different superscript letters are significantly different (*p* < .05). L*, a* and b* denote luminance, red‐greenness and yellow‐blueness, respectively

The HMT induced a Maillard reaction in the mixed flour, which significantly impacted the appearance and odor quality of the sweet dumplings. Additionally, the beans themselves contained a soybean odor that further affected the overall aroma. Therefore, we had determined which sweet dumpling possessed the most similar characteristics to the G‐SD by examining the variation in color of the dumplings and the primary components identified by the electronic nose (refer to Table 5 and Figure 5). The decrease in L* values and the significant increase in a* and b* values indicated a reduction in the level of whiteness and an increase in the levels of redness and yellowness of the blended powders. Under high temperature and humidity conditions, the mixed powder may have undergone the Maillard reaction, resulting in a browning color, as supported by Singh et al. (2011). Legumes, which give sweet dumplings their distinct smell, volatilized. The majority of the gaseous components were made up of aldehydes, sulfides, and hydroxyls. An electronic nose sensor array was created using an appropriate gas sensor to analyze how the smell of sweet dumplings changed before and after steaming. The smell analysis module primarily used PCA to provide a more intuitive representation of the study results. Given that the combined contribution values of A and B had exceeded 85% (Figure 5), PC1 and PC2 had been considered representative samples of the primary informative features of volatiles in sweet dumplings (Van Ruth et al., 2004). The R‐SD's odor contribution values before and after steaming had been close to those of the G‐SD, indicating that the addition of red adzuki beans had not caused significant changes in the quality of the sweet dumplings. Conversely, the B‐SD's odor contribution values showed significant changes before and after steaming. This may have been due to the higher fat content in black beans undergoing a more severe Maillard reaction under high temperature and humidity, leading to a significant increase in volatile flavor compounds and consequently reducing the quality of the sweet dumplings.

## 4 CONCLUSIONS

The incorporation of legumes into GRF combined with HMT significantly affects the quality of sweet dumplings and the properties of the composite flour. Among the samples tested, R‐GRF and R‐SD demonstrated the lowest eGI values, at 52.01 and 52.21, respectively. Structural analysis revealed that these samples had a higher starch crystal content, intact granules, and a compact, stable structure, effectively inhibiting amylase access and reducing the starch hydrolysis rate, thereby supporting the lower eGI values. Thermal performance assessments indicated that the inclusion of red beans in GRF and the synergistic application of HMT played critical roles in starch gelatinization, thermal stability, and expansion. This process also facilitated the formation of protein–starch complexes, significantly reducing amylase sensitivity and increasing resistant starch content. A comprehensive quality evaluation of the sweet dumplings revealed that R‐SD closely resembled G‐SD in color and aroma, while surpassing it in texture, taste, and other quality attributes. The quality of the product is closely tied to the processing techniques, warranting further research and optimization of the processing methodology for R‐SD.

## AUTHOR CONTRIBUTIONS

**Sun Qiangqiang:** Writing – original draft (equal). **Liu Xuhua:** Data curation (equal). **Yan Rong:** Visualization (equal). **Dong Qiyun:** Supervision (equal). **Yang Liping:** Supervision (equal).

## FUNDING INFORMATION

This work was supported by the Bengbu Science and Technology Project [2022ny04].

## CONFLICT OF INTEREST STATEMENT

The authors declare that they have no known competing financial interests which could influence the work reported in this paper.

## Data Availability

We declare that the data in this paper come from our experiments, which are reliable. The dataset analyzed during the current study is available from the corresponding author on reasonable request.
